# Clade Distinction and Tracking of Clonal Spread by Fourier‐Transform Infrared Spectroscopy in Multicenter *Candida* (*Candidozyma*) *auris* Outbreak

**DOI:** 10.1111/myc.70085

**Published:** 2025-07-04

**Authors:** Camylla C. de Melo, Halana L. N. L. de Oliveira, Bruna R. Souza, Carla V. R. Moura, Rodrigo Oliveira, Rafael W. Bastos, Karoline Kristina Kemmerich, João N. de Almeida‐Júnior, Arnaldo Lopes Colombo, Bram Spruijtenburg, Jacques F. Meis, Patrice Le Pape, Carolyn G. J. Moonen, Reginaldo G. de Lima‐Neto

**Affiliations:** ^1^ Laboratory for Research and Diagnosis of Tropical Diseases, Center for Medical Sciences Federal University of Pernambuco (UFPE) Recife Brazil; ^2^ Post‐Graduation Program in Tropical Medicine Federal University of Pernambuco (UFPE) Recife Brazil; ^3^ Bruker Daltonics GmbH & Co. KG Bremen Germany; ^4^ Department of Microbiology and Parasitology Federal University of Rio Grande Do Norte Natal Brazil; ^5^ Department of Medicine Federal University of São Paulo São Paulo Brazil; ^6^ Antimicrobial Resistance Institute of São Paulo (ARIES) São Paulo Brazil; ^7^ Radboudumc – CWZ Center of Expertise for Mycology Nijmegen the Netherlands; ^8^ Department of Medical Microbiology and Immunology Canisius‐Wihelmina Hospital (CWZ)/Diccon Nijmegen the Netherlands; ^9^ Institute of Translational Research, Cologne Excellence Cluster on Cellular Stress Responses in Aging‐Associated Diseases (CECAD) University of Cologne Cologne Germany; ^10^ Cibles et Médicaments Des Infections et de L'immunité, IICiMed, UR 1155 Université de Nantes Nantes France

**Keywords:** *Candida auris*, clades, FT‐IR spectroscopy, typing

## Abstract

**Background:**

*Candida* (*Candidozyma*) *auris* has distinct genetic clades. Clade distinction is relevant for infection control and epidemiological purposes. State‐of‐the‐art typing methodologies for clade distinction are based on genomic approaches, such as short tandem repeat (STR) analysis and whole‐genome sequencing (WGS). However, they are time‐consuming and expensive. Fourier transform infrared spectroscopy (FT‐IR) is an alternative tool for strain typing based on their unique biochemical spectral profiles.

**Objectives:**

To apply FT‐IR to differentiate *C. auris* clades and evaluate epidemiological relationships based on biochemical data among isolates from a multicenter *C. auris* outbreak in the state of Pernambuco, northeastern Brazil.

**Methods:**

Sixty‐nine *C. auris* strains from clades I, II, III, and IV were analysed. Fifty‐nine were clade IV strains obtained from three outbreaks that took place in Pernambuco state, northeastern Brazil. An adjusted FT‐IR spectroscopy protocol was applied to obtain carbohydrates and lipid fingerprints. Short Tandem Repeat (STR) analysis was used in order to validate the spectroscopy approach.

**Results:**

The adjusted preparation protocol for FT‐IR analysis improved the spectral quality by 31.42% compared to standard protocol. FT‐IR allowed us to discriminate *C. auris* clades I to IV. Moreover, important similarities were observed in *C. auris* clade IV strains obtained from two of the three hospitals, based on polysaccharides (1300–800 cm^−1^) plus lipids (3000–2800 cm^−1^ and 1500–1400 cm^−1^) spectra. STR confirmed the similarity results obtained by FT‐IR, clustering the strains from two different hospitals.

**Conclusions:**

The IR Biotyper is fast, easy‐to‐use, and a promising alternative for moderate‐to‐high‐complexity laboratories to differentiate *C. auris* clades. Furthermore, this technique has the potential for isolate‐level source tracking, which could be valuable for monitoring transmission routes in clinical settings.

## 1 Introduction

*Candida auris* (*Candidozyma auris*) is an emerging multidrug‐resistant yeast responsible for invasive infections with high mortality rates, representing a serious global health threat [1]. Since its first recognition, *C. auris* has caused numerous outbreaks worldwide, with infections that are often difficult to control due to emerging antifungal resistance and capacity to persist in healthcare settings. The World Health Organisation (WHO) has categorised *C. auris* as a critical priority pathogen in its fungal priority pathogens list, underscoring the urgent need for research, development, and public health action to combat its spread [2].

This emerging pathogen is known for the high genetic diversity and can be separated into distinct phylogenetic clades, displaying peculiarities of geographic distribution, virulence, and resistance [3, 4, 5]. Until now, six clades have been classified: South Asian (clade I), East Asian (clade II), African (clade III), South American (clade IV), Iranian (clade V), and Indomalayan (clade VI). Among these, Clade I, with a worldwide distribution, exhibits high resistance rates, with over 90% of isolates resistant to fluconazole, over 50% resistant to amphotericin B, and 5% resistance to echinocandins [6]. Clade II, limited to Japan and South Korea, contrasts with Clade I in its lower resistance rates and is not commonly associated with hospital outbreaks. Clade III, apart from first being recognised in South Africa, has been linked to outbreaks in Spain and the United States [6]. Clade IV has been mainly reported in Latin America and the United States, with up to 30% resistance to amphotericin B among Colombian strains [7], as well as a propensity for high biofilm formation [8]. Although Clade V has been identified in Iran and Clade VI in Singapore, they are not included in this study.

In Brazil, *C. auris* has emerged as a pressing concern, particularly in the state of Pernambuco, where 68 of the 90 reported Brazilian cases where detected by 2024. Following the first outbreak in Pernambuco in 2022, the Brazilian National Health Surveillance Agency released a revised version of the technical note focused on identification, prevention and control of *C. auris* infections in healthcare facilities [9]. Current laboratory practices for identifying *C. auris* typically involve either rDNA sequencing or MALDI‐TOF MS. Although effective for species‐level identification, ITS sequencing may have limitations in rapidly differentiating clades, which could facilitate outbreak management in real‐time clinical settings. At present, short tandem repeat (STR) typing and whole‐genome sequencing (WGS) are the typing approaches with the most discriminatory power, and essential for tracing the origins of nosocomial transmission [10, 11]. However, both methodologies are time‐consuming, costly, and require complex data analyses tools and expertise. The IR Biotyper as a tool in bacterial nosocomial outbreak investigations has been ample investigated in recent years [12, 13, 14] while its use in fungal outbreaks has been lagging behind [15, 16, 17, 18].

To address these challenges, this study investigated the use of the IR Biotyper for distinguishing *C. auris* clades [18, 19]. The IR Biotyper utilises Fourier‐transform infrared (FT‐IR) spectroscopy to differentiate microbial isolates based on spectral profiles that reflect their biochemical composition. Implementing such rapid tools could provide critical insights into transmission patterns in outbreaks and offers a valuable complementation to current sequencing‐based approaches in public health responses to *C. auris* . Here, we explore its potential to differentiate the four major clades of *C. auris* (Clades I–IV), assessing its utility as a fast and reliable method for clade differentiation and potential strain typing. Although we are certain of the potential applicability of FT‐IR for Clades V and VI, we focused this study on the most prevalent Clade in South America.

## 2 Materials and Methods

### 2.1 Site of Study and Ethical Considerations

**FIGURE 1:**
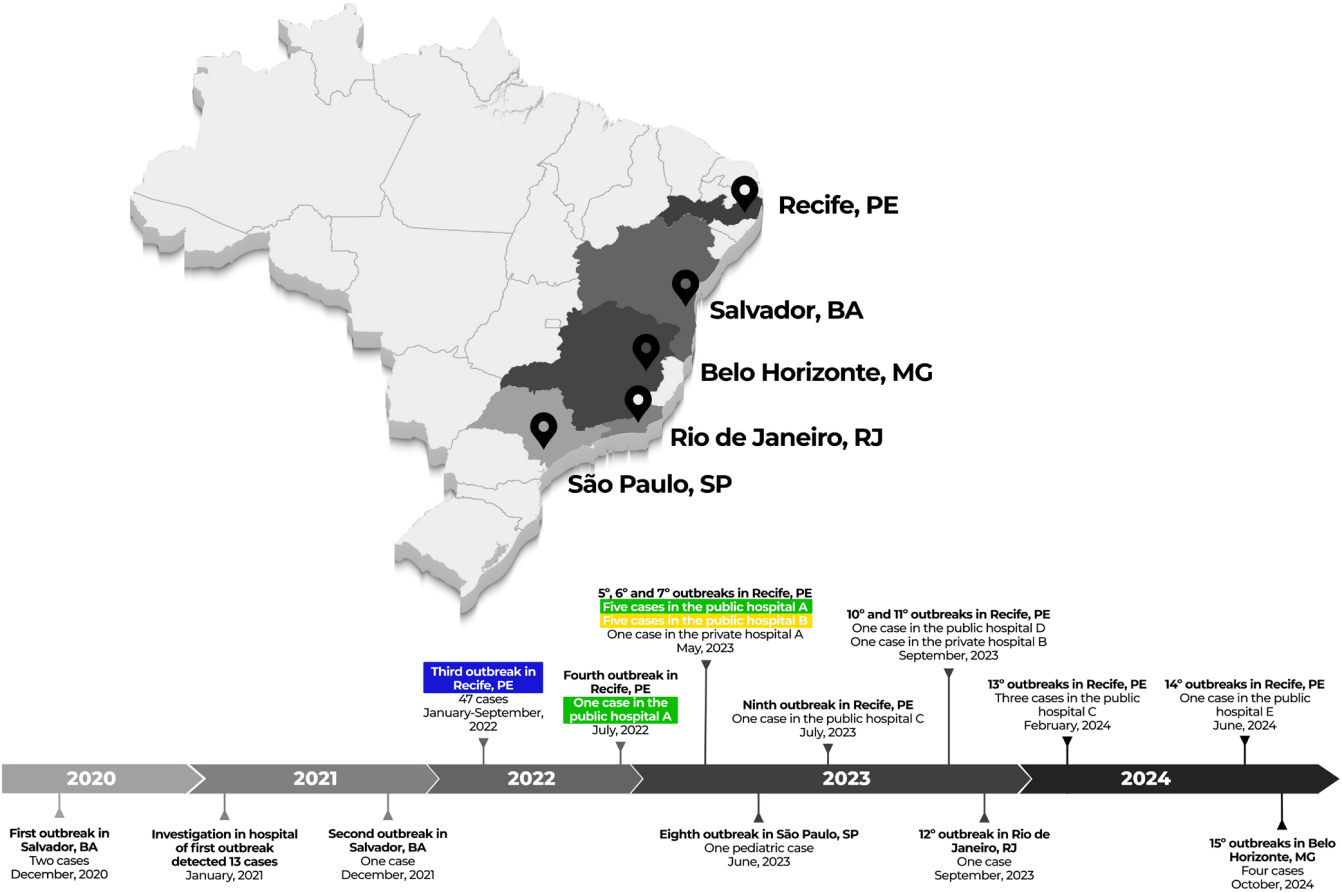
Timeline of outbreaks related to *Candida auris* in Brazil. Outbreaks were timed according to risk alerts issued by Brazilian National Surveillance Sanitary Agency or State Health Departments.

Sixty‐nine *C. auris* strains from clades I, II, III and IV were included in this study as summarised in Table 1. The Brazilian *Candida auris* strains (*n* = 59) were obtained from outbreaks that took place in three Tertiary Public Hospitals in Pernambuco, Northeastern Brazil. Forty‐seven strains were obtained from the hospital abbreviated as HR, seven from the hospital abbreviated as HTRI, and five from the hospital abbreviated as HMA. These three hospitals are part of the Pernambuco State health department and are highlighted in blue, yellow and green, respectively, in Figure 1.

**TABLE 1:** Distribution of *Candida auris* strains analysed per Clade and respective numbers of spectra obtained by IR Biotyper.

Clade	Origin isolate	Isolates	IRBT spectra
Clade I	Kuwait	2	56
Clade II	CDC B11220	1	28
Clade III	Spain	1	28
Clade IV	Brazil	59	1279
	CDC B11903 (USA)	1	36
	Colombia	3	95
	Venezuela	2	60
**Total**		**69**	**1582**

This study was submitted to the Human Research Ethics Committee of the Center for Health Sciences at the Federal University of Pernambuco (UFPE, Brazil) under protocol CAAE 66736422.5.0000.5208 and received approval under protocol number 6.011.801.

### 2.2 Strain Identification

The identification of *C. auris* isolates to the species *taxon* was performed in the Laboratory for Research and Diagnosis of Tropical Diseases at the UFPE, using MALDI‐TOF MS Biotyper (Autoflex III Smartbean, Bruker Daltonics, Bremen, Germany), according to the manufacturer's recommendation. The spectra were obtained in triplicate and exported to the database MALDI Biotyper (Biotyper system, version 3.1, Bruker Daltonics Inc., USA/Germany) [15]. Furthermore, ITS rDNA sequencing analyses were conducted with previously described primers and conditions [4, 20].

### 2.3 IR Biotyper Analysis

#### 2.3.1 Culture Conditions

*C. auris* isolates were cultured on Sabouraud dextrose agar (Plastlabor, Rio de Janeiro, Brazil). Each isolate was incubated at 37°C for 24 h to control for pure culture and to achieve confluent growth, which is crucial for the sample's spectral consistency and quality. To ensure robustness and account for biological variability, each isolate was sub‐cultured and analysed on three independent days, yielding three biological replicates of each *C. auris* isolate.

#### 2.3.2 Sample Preparation and Spectrum Acquisition

Following incubation, an adjusted preparation protocol previously published by Contreras and Morgan [16] was used, because the standard protocol yielded poor‐quality spectra. The key modifications are related to biomass (one 10 μL loopful instead of two), and to the volume of suspension plated before spectra acquisition (10 μL instead of 15 μL). Accordingly, a full 10 μL loop of the confluent part of the *C. auris* culture was harvested. The biomass was then transferred into an IR Biotyper suspension vial containing metal beads (IR Biotyper kit, Bruker Daltonics GmbH & Co. KG, Bremen, Germany) and 70 μL of 70% ethanol. This suspension was vortexed for 1 min to ensure thorough homogenization and suspended with 70 μL deionised water. For each strain, five technical replicates of 10 μL were pipetted onto a reusable 96‐well silicon IR Biotyper plate (Bruker Daltonics GmbH & Co. KG, Bremen, Germany) and allowed to dry at room temperature. Once dry, the IR Biotyper measurements were performed. Spectra were acquired (transmission mode between wave numbers 4000–500 cm^−1^) and processed by OPUS software V.8.2.28 (Bruker Optics, Ettlingen, Germany) on an IR Biotyper with the corresponding IR Biotyper software V4.0 (Bruker Daltonics GmbH & Co. KG, Bremen, Germany) for data analysis. During measurement, the IR Biotyper software checks each spectrum obtained per spot. This check is called a quality test (abbreviated as QT). If the spectra are of poor quality, (e.g., due too low absorption values), the QT will fail.

#### 2.3.3 Spectral Analysis

For clade differentiation analysis, only specific, biologically relevant wavenumber regions of the spectrum were selected to focus on informative molecular components, improving classification accuracy. The selection of these regions is processed in the IR Biotyper software using a method known as “splicing,” which allows the exclusion of non‐informative regions and enhances targeted differentiation by focusing on spectral areas that contribute most effectively to clade separation. Two splicing methods were applied to assess the best approach for each step of the study. For *C. auris* clade differentiation, the default splicing method was used which targets the polysaccharide region between 1300 and 800 cm^−1^. For strain typing, an adjusted splicing method combining the default polysaccharide region (1300–800 cm^−1^) with additional regions that target lipid components: 3000–2800 cm^−1^ and 1500–1400 cm^−1^ was used. This adjusted splicing method was used as these additional lipid profiles decreased spectral variability and enhanced geographical differentiability.

#### 2.3.4 Data Analysis

Spectral analysis and data visualisation were performed using Linear Discriminant Analysis (LDA) available in the IR Biotyper software, with isolate ID set as the target group. This analysis generated 2D and 3D plots capturing up to 100% of the variance using a maximum of 40 principal components. To visualise differences across all principal components, deviation plots were created, where the solid line represents the mean spectrum, and the shaded area indicates the standard deviation.

### 2.4 STR Assay

In order to validate the infrared typing results, STR genotypes of the Brazilian *C. auris* outbreak were obtained from a previous publication [21] in collaboration with the Special Mycology Laboratory at the Federal University of São Paulo. Colombian clade IV strains, the CDC B11903 clade IV reference strain from the USA, and two strains each of clades I, II, III, V, and VI were included as control.

## 3 Results

The MALDI‐TOF MS tool reliably identified all isolates included in this study at the species level as *C. auris* , with scores above 2.0. Genomic analysis also identified the isolates as *C. auris* , and Bayesian inference (GTR + G) was the best model for ITS alignment. As shown by the dendrogram, the strains from Pernambuco, Brazil are closely related to *C. auris* strains L481/2015 from Venezuela, 2MG‐A020357 from Israel, and 20‐1498 from Mexico, all classified as belonging to the Clade IV (Figure S1). The sequences of the isolates were deposited in GenBank, and the accession numbers are provided in Table S1.

A total of 69 *Candida auris* isolates from Clades I, II, III, and IV were analysed, generating 1582 IR Biotyper spectra. After implementing of the adjusted protocol, 90.1% were QT passed, and thus of good quality. An improvement of 31.4% compared to standard protocol that reached 58.7% of QT passed. The adjusted protocol produced better results mainly in two sample quality control parameters: absorption and fringes. Clade I included 2 isolates from Kuwait, producing 56 spectra. Clade II, consisted of a single isolate from Japan (CDC B11220), yielding 28 spectra. One Clade III isolate from Spain gave 28 spectra. Clade IV, representing the majority of isolates, included 59 isolates from Brazil (Pernambuco) with 1279 spectra, 3 isolates from Colombia with 95 spectra, and 2 isolates from Venezuela with 60 spectra. Finally, one Clade IV reference strain from the CDC (B11903) with 36 spectra was included in this study (Table 1).

**FIGURE 2:**
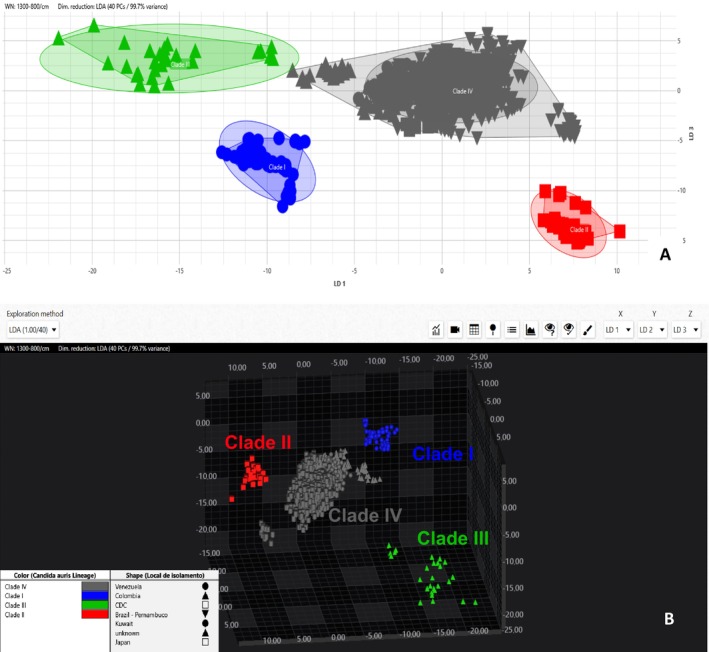
Scatter plots where each point represents a spectrum, with different colours indicating the clades showing Clade I isolates in blue, Clade II isolates in red, Clade III in green, Clade IV in grey. Shapes of the points depict the country of origin of each isolate. Total of 1582 spectra are displayed. Graphs and legend created with the IR Biotyper software. (A) 2D visualisation of *Candida auris* clades (LDA 40 PCs, 99.75% variance, target group = isolate ID). X‐axis is depicting LD1, y‐axis is depicting LD2, together displaying 74.48% variance. (B) 3D visualisation of *Candida auris* clades (LDA 40 PCs, 99.7% variance, target group = isolate ID). X‐axis displays LD1, y‐axis displays LD2, z‐axis displays LD3, together displaying 85.19% variance.

The first objective of this study was to explore the possibility of *Candida auris* clades differentiation using the IR Biotyper. Figure 2A,B, display 2D and 3D scatter plots, respectively, depicting the clear separation of the four different clades. Figure S2 displays a 3D plot which is interactive and can be rotated, shown as four separate snapshots. These snapshots provide a static look from multiple angles visualising the separation and clustering of clades more clearly.

As the majority of isolates included in this study originated from *C. auris* Clade IV, a further investigation was performed on solely isolates originating from geographical locations in South America, including Venezuela, Colombia, Brazil as well as a reference strain from the CDC/Atlanta. The objective of the second part of this study was to explore the possibly of differentiation of isolates from various geographical regions within clade IV. In total, this analysis included 1470 spectra from 65 *C. auris* clade IV isolates. Figure S3 displays the 3D scatter plot, where the colours depict geographical origin. Accordingly, the red spectra represent isolates from Brazil, grey from Venezuela, green from the CDC reference strain, and blue for isolates from Colombia. Interestingly, the Colombian isolates show slight differences compared to the others, although they do not form a separate cluster.

Within Clade IV isolates, the majority of isolates originate from Brazil, specifically from patients admitted in 3 different hospitals in Pernambuco state—HR, HTRI, and HMA (Figure 1). The third objective of this study was to explore the possibility to perform source tracking of these 59 clinical isolates from 1279 spectra obtained from these three hospitals. In the Figure S4 presented as a 3D scatter plot, a clear distinct cloud of HMA samples (green) and a clustering of HR and HTRI (blue and yellow, respectively) is observed. As 3D plots display only three dimensions (LD1, LD2, LD3), a parallel plot is shown displaying all 30 LDA dimensions where a clear overlap of HR and HTRI isolates is observed. Interestingly, the HR and HTRI samples are mixed within the same cloud. Notably, two highly related genotypes were found in isolates from Brazil by STR dendrogram (Figure S5). In the end of the dendrogram dedicated to Brazilian strains, HMA forms a cluster, and HR and HTRI are mixed in another large cluster.

## 4 Discussion

The increase and global spread of *C. auris* in the last decade is concerning, given the ability of this organism to cause prolonged outbreaks in health care facilities, the extent of resistance to treatment and disinfectant agents, besides the high mortality rates in invasive infections. Resistance to at least one class of antifungals is frequently observed, and such resistance levels change substantially according to areas and settings, the time of hospital introduction of the pathogen, mainly due to clade distribution [3]. The clades present different behaviours, and identifying them correctly and quickly may guide infection control measures, antimicrobial stewardship programmes, epidemiological surveillance actions, and understanding the dynamics of regional and global dissemination.

More research is needed to develop rapid and affordable identification and typing. In Brazil, The Brazilian National Surveillance Sanitary Agency has published and revised a technical note, which instructed the Infection Control Services and Clinical Laboratories that the gold standard for *C. auris* identification is based on ITS sequence analysis or mass spectrometry [9]. The microbial identification workflow in our laboratory is based on MALDI‐TOF MS and ITS sequencing. However, both methodologies do not provide sufficient resolution to differentiate *C. auris* isolates to the correct clade, so we explored successfully the possibility of differentiating with the IR Biotyper.

To the best of our knowledge, the study published in 2020 by Vatanshenassan et al. [15] was the first to apply Fourier‐Transform Infrared spectroscopy for typing *Candida auris*. The five typing protocols (MLST, ITS sequencing, AFLP, MALDI‐TOF and IR Biotyper) showed MLST analysis as the preferred method for evaluating *C. auris* outbreaks. The better agreement with this method was sequencing of ITS (45% similarity), followed by typing by IR‐BT (33% similarity). The researchers conclude that FT‐IR spectroscopy requires further optimization and evaluation before it can be used as an epidemiological typing method, as a rapid method for tracing nosocomial fungal outbreaks. Our study shows that protocol modifications may improve the discriminatory power of FT‐IR spectroscopy for strain typing.

In the first step of our study, we tested a fine‐tuned preparation protocol to optimise conditions for creating high‐quality FT‐IR spectra of *Candida auris* with good absorption values, focusing on the sample preparation method. This protocol, adapted after Contreras and Morgan [16], effectively differentiated between *C. auris* Clade I, II, III, and IV. By adding third‐dimension scatter plots (Figures 2 and S2), we gained additional insight into the clustering and separation of *C. auris* clades, providing an even clearer view of the differentiation. We are aware that the small number of isolates from Clades I, II, and III is a limitation; however, we also believe that the result obtained is a relevant step towards the future consolidation of a new methodology for distinguishing Clades. The study by Contreras and Morgan [16] also showed that the IR Biotyper was able to separate *Candida auris* (*n* = 28) into two clusters. The weakness of this study lies in the fact that only two clades, Clades I and III, were used.

In the second part of the study, we looked at the differentiation of *Candida auris* clade IV isolates from various locations. Specifically, we included isolates from Brazil (Pernambuco state), Venezuela, and Colombia, as well as a reference strain from the CDC. Interestingly, the colour‐coded 3D scatter plot by geographical origin displayed that the Colombian isolates show slight differences compared to the others, although they did not form a completely separate cluster (Figure S3). These spectral differences may be associated with resistance profiles as the Brazilian isolates were susceptible to all tested antifungal drugs [21]. However, Colombian isolates were resistant to both fluconazole and amphotericin B. The method to evaluate the antifungal profile of all strains was broth microdilution by EUCAST. These variations in drug resistance may correlate with slight biochemical differences detectable in the spectra which has been suggested previously also with echinocandin resistant *C. auris* [22]. It has been shown that amphotericin B‐resistant *C. auris* strains have different contents of sphingolipids, such as glucosylceramide, phytoceramide and dihydroceramides [23]. Interestingly, a study published by Stieber et al. [24] showed that inhibition of myorocin‐mediated sphingolipid synthesis reversed the resistance of *C. auris* , making them amphotericin B‐susceptible. However, the same inhibitor showed limited effect on fluconazole susceptibility. In our spectral analyzes, we used a default splicing method plus lipid profile, which we named adjusted splicing method. This optimization that produced an improvement in geographic distinction was found during the study. A significant limitation of our study is the small number of isolates included from outside Brazil. It will be essential to expand the dataset with more isolates from different geographical areas and clades to further explore these differences.

In the third and last part of the study presented here, we focused on the source tracking by phenotypic relationships of the yeasts isolated from three hospitals in Pernambuco, Brazil—specifically, HR, HTRI, and HMA, highlighted respectively in blue, yellow, and green in Figure 1. In Figure S4, we can see a distinct cloud of HMA patient samples (green) and a combined cloud of HTRI and HR samples (yellow and blue). Interestingly, the HTRI and HR samples are mixed within the same cloud, suggesting they are indistinguishable on the basis of the composition of carbohydrates and lipids detected by spectral analysis, consistent with clonal spread. This is plausible given that patients are often transferred between these two hospitals. This clustering of HR and HTRI isolates supports the hypothesis that patient movement between these hospitals played a significant role in the spread of *C. auris*. These Biotyper results were confirmed by STR analysis, where HMA strains formed a single cluster. The HTRI strains are among those of the HR. Several methodologies to genotype *C. auris* isolates may be applied, each with its own advantages and disadvantages. STR analysis is ideal for laboratories that do not have WGS capacity, and its implementation in outbreak environments by *C. auris* has already exhibited its value in an epidemiological context for understanding potential relationships between clinical strains [25, 26].

A recently published study investigated the phylogenetic relationships among 56 *C. auris* clinical strains during an outbreak in a tertiary university hospital in Italy [18]. A total of 855 spectra were obtained and two distinct clusters were observed by dendrogram and distance matrix analysis. After the first positive case in the ICU ward, the hospital established a surveillance program that detected 55 more cases during the protracted 14‐month‐outbreak. The authors applied FT‐IR using IR Biotyper to cluster all 56 *C. auris* strains to the same group or not. The innovation of their results consists in the definition of a clustering cut‐off with statistical–mathematical approach. Authors argue that this study is based on phylogenetic relationships, and although two very distinct clusters were obtained, the study does not use genomic methods to confirm the results obtained through FT‐IR, since the *C. auris* strains were identified only by MALDI‐TOF MS.

As mentioned earlier as a limitation of our study, the analysis of more well‐characterised strains from different clades is essential to consolidate our results and allow the creation of a classifier. In this sense, an interesting study published recently [19] combined FT‐IR and artificial neural networks with the aim of classifying a set of 74 *C. auris* isolates belonging to the five clades (I–V). The classifier proposed allowed us to discriminate the four primary *C. auris* clades (I–IV) with a precision of 96%. Our future work will focus on developing a classifier for automated differentiation of *C. auris* clades after obtaining more spectra of underrepresented clades in the current dataset.

FT‐IR may prove vital for hospital infection control, both for being able to distinguish clades, and for characterising yeasts at the lineage level.

## 5 Conclusion

In this study, we tested an adjusted preparation protocol to optimise conditions for generating high‐quality FT‐IR spectra with improved absorption values, focusing on the sample preparation method. The IR Biotyper successfully differentiated between *C. auris* clades I, II, III, and IV, offering high resolution, fast, and cost‐effective clade differentiation. Furthermore, we confirmed the high similarity between *C. auris* clade IV strains from two hospitals as previously shown by genotyping.

The IR Biotyper presents a promising alternative for moderate to high‐complexity laboratories seeking an accessible, moderately priced strain‐typing platform.

## Author Contributions

**Camylla C. de Melo:** investigation, methodology, formal analysis, data curation. **Halana L. N. L. de Oliveira:** investigation, methodology, formal analysis, data curation. **Bruna R. Souza:** investigation, methodology, formal analysis, data curation. **Carla V. R. Moura:** investigation, methodology, formal analysis, data curation. **Rodrigo Oliveira:** conceptualization, methodology, validation, writing – review and editing. **Rafael W. Bastos:** conceptualization, validation, methodology, writing – review and editing. **Karoline Kristina Kemmerich:** investigation, methodology, formal analysis, data curation. **João N. de Almeida‐Júnior:** conceptualization, methodology, validation, writing – review and editing. **Arnaldo Lopes Colombo:** conceptualization, methodology, validation, writing – review and editing. **Bram Spruijtenburg:** investigation, methodology, formal analysis, data curation. **Jacques F. Meis:** conceptualization, methodology, validation, writing – review and editing. **Patrice Le Pape:** conceptualization, methodology, validation, writing – review and editing. **Carolyn G. J. Moonen:** conceptualization, investigation, methodology, formal analysis, validation, data curation, writing – original draft, writing – review and editing. **Reginaldo G. de Lima‐Neto:** conceptualization, investigation, methodology, formal analysis, validation, data curation, writing – review and editing, writing – original draft, funding acquisition.

## Conflicts of Interest

The authors declare no conflicts of interest.

## Supporting information


**Figure S1.** Maximum likelihood (ML) tree obtained using ITS rDNA sequences from *Candida* species. Study species are in bold. Maximum likelihood (ML‐BS) bootstrap and Bayesian inference posterior probability (BPP) of 70% and 0.90 respectively, are shown near the nodes. The tree was rooted in *Cryptococcus gattii* VGI (CBS 6289). The bar represents the expected number of replacements per site. The superscript * indicates the ex‐type strain.

**Figure S2.** Four snapshots of different angles from the *Candida auris* clades obtained by 3D scatter plots (LDA 40 PCs, 99.7% variance, target group = isolate ID) showing Clade I in blue, Clade II in red, Clade III in green, and Clade IV in grey. X‐axis displays LD1, y‐axis displays LD2, z‐axis displays LD3. Each dot/shape represents one spectrum. Total of 1582 spectra are displayed. Shapes depict country of origin of the isolated. Graph and legend created with the IR Biotyper software.

**Figure S3.** 3D scatter plot of *Candida auris* Clade IV analysed depicting different geographical origins (LDA 40 PCs, 99.6% variance, target group = isolate ID). Blue: Colombia; Grey: Venezuela; Red: Brazil; Green: CDC reference strain. X‐axis displays LD1, y‐axis displays LD2, z‐axis displays LD3, together displaying 55.73% variance. Each dot/shape represents one spectrum. Total of 1470 spectra are displayed. Graph created with the IR Biotyper software. Adjusted splicing method: 1300–800 cm^−1^ (polysaccharide region), 3000–2800 cm^−1^ (CH region), 1500–1400 cm^−1^ (2nd fatty acid).

**Figure S4.** 3D scatter plot of *Candida auris* Clade IV from the state of Pernambuco, Brazil, depicting patient isolates originate from 3 different hospitals (LDA 40 PCs, 99.6% variance, target group = isolate ID). Green: HMA; Blue: HR, Yellow: HTRI. X‐axis displays LD1, y‐axis displays LD2, z‐axis displays LD3, together displaying 58.69% variance. Each dot/shape represents one spectrum. Total of 1279 spectra are displayed. Bottom left of the figure is 2D visualisation of Pernambuco subset *Candida auris* strains. Graph created with the IR Biotyper software. Adjusted splicing method: 1300–800 cm^−1^ (polysaccharide region) + 3000–2800 cm^−1^ (CH region) + 1500–1400 cm^−1^ (2nd fatty acid).

**Figure S5.** STR genotypes of Brazilian *C. auris* isolates typed with five multiplex PCRs, M2, M3‐I, M3‐II, M3‐III, and M9, which amplify 14 STR targets with repeat sizes of 2, 3, or 9 nucleotides. Cluster analysis displayed that clinical strains from HMA formed a distinct cluster in the end of dendrogram.

**Table S1.** Overview of *Candida* spp. used in this study. All strains were used to construct the ITS phylogenetic analysis. The strains highlighted in bold were used in Fourier‐Transform Infrared Spectroscopy analysis.

## Data Availability

All relevant data are within the paper and its Supporting Information file with Genbank accession numbers. The data that support the findings of this study are available from the corresponding author upon reasonable request.

## References

[1] J. F. Meis and A. Chowdhary , “ *Candida auris*: A Global Fungal Public Health Threat,” Lancet Infectious Diseases 18, no. 12 (2018): 1298–1299. 30293876 10.1016/S1473-3099(18)30609-1

[2] H. Y. Kim , T. A. Nguyen , S. Kidd , et al., “ *Candida auris* – A Systematic Review to Inform the World Health Organization Fungal Priority Pathogens List,” Medical Mycology 62, no. 6 (2024): myae042. 38935900 10.1093/mmy/myae042 PMC11210622

[3] S. R. Lockhart , K. A. Etienne , S. Vallabhaneni , et al., “Simultaneous Emergence of Multidrug‐Resistant *Candida auris* on 3 Continents Confirmed by Whole‐Genome Sequencing and Epidemiological Analyses,” Clinical Infectious Diseases 64, no. 2 (2017): 134–140. 27988485 10.1093/cid/ciw691 PMC5215215

[4] B. Spruijtenburg , H. Badali , M. Abastabar , et al., “Confirmation of Fifth *Candida auris* Clade by Whole Genome Sequencing,” Emerging Microbes & Infections 11, no. 1 (2022): 2405–2411. 36154919 10.1080/22221751.2022.2125349 PMC9586689

[5] C. Suphavilai , K. K. K. Ko , K. M. Lim , et al., “Detection and Characterisation of a Sixth *Candida auris* Clade in Singapore: A Genomic and Phenotypic Study,” Lancet Microbe 5, no. 9 (2024): 100878. 39008997 10.1016/S2666-5247(24)00101-0

[6] N. A. Chow , J. F. Muñoz , L. Gade , et al., “Tracing the Evolutionary History and Global Expansion of *Candida auris* Using Population Genomic Analyses,” MBio 11, no. 2 (2020): e03364‐19. 32345637 10.1128/mBio.03364-19 PMC7188998

[7] E. Misas , P. L. Escandón , L. Gade , et al., “Genomic Epidemiology and Antifungal‐Resistant Characterization of *Candida auris*, Colombia, 2016–2021,” mSphere 9, no. 2 (2024): e0057723. 38299868 10.1128/msphere.00577-23 PMC10900874

[8] C. C. de Melo , B. R. de Sousa , G. L. da Costa , M. M. E. Oliveira , and R. G. de Lima‐Neto , “Colonized Patients by *Candida auris*: Third and Largest Outbreak in Brazil and Impact of Biofilm Formation,” Frontiers in Cellular and Infection Microbiology 13 (2023): 1033707. 36756619 10.3389/fcimb.2023.1033707 PMC9900136

[9] Ministry of Health Brazil , “Guidelines for Identification, Prevention and Control of *Candida auris* Infections in Health Services,” (2022), https://www.gov.br/anvisa/pt‐br/centraisdeconteudo/publicacoes/servicosdesaude/notas‐tecnicas/2020/nota‐tecnica‐gvims‐ggtes‐anvisa‐no‐02‐2022‐revisada‐em‐07‐10‐2022/view.

[10] B. Spruijtenburg , J. F. Meis , P. E. Verweij , T. de Groot , and E. F. J. Meijer , “Short Tandem Repeat Genotyping of Medically Important Fungi: A Comprehensive Review of a Powerful Tool With Extensive Future Potential,” Mycopathologia 189, no. 5 (2024): 72. 39096450 10.1007/s11046-024-00877-8 PMC11297828

[11] T. de Groot , B. Spruijtenburg , L. A. Parnell , N. A. Chow , and J. F. Meis , “Optimization and Validation of *Candida auris* Short Tandem Repeat Analysis,” Microbiology Spectrum 10, no. 5 (2022): e0264522, 10.1128/spectrum.02645-22. 36190407 PMC9603409

[12] D. Martak , B. Valot , M. Sauget , et al., “Fourier‐Transform InfraRed Spectroscopy Can Quickly Type Gram‐Negative Bacilli Responsible for Hospital Outbreaks,” Frontiers in Microbiology 10 (2019): 1440. 31293559 10.3389/fmicb.2019.01440 PMC6606786

[13] N. Rakovitsky , S. Frenk , H. Kon , et al., “Fourier Transform Infrared Spectroscopy Is a New Option for Outbreak Investigation: A Retrospective Analysis of an Extended‐Spectrum‐Beta‐Lactamase‐Producing *Klebsiella pneumoniae* Outbreak in a Neonatal Intensive Care Unit,” Journal of Clinical Microbiology 58, no. 5 (2020): e00098‐20. 32161093 10.1128/JCM.00098-20 PMC7180251

[14] G. Uribe , S. J. Salipante , L. Curtis , et al., “Evaluation of Fourier Transform‐Infrared Spectroscopy (FT‐IR) as a Control Measure for Nosocomial Outbreak Investigations,” Journal of Clinical Microbiology 61, no. 10 (2023): e0034723. 37787542 10.1128/jcm.00347-23 PMC10595069

[15] M. Vatanshenassan , T. Boekhout , N. Mauder , et al., “Evaluation of Microsatellite Typing, ITS Sequencing, AFLP Fingerprinting, MALDI‐TOF MS, and Fourier‐Transform Infrared Spectroscopy Analysis of *Candida auris* ,” Journal of Fungi 6, no. 3 (2020): 146, 10.3390/jof6030146. 32854308 PMC7576496

[16] D. A. Contreras and M. A. Morgan , “Surveillance Diagnostic Algorithm Using Real‐Time PCR Assay and Strain Typing Method Development to Assist With the Control of *C. auris* Amid COVID‐19 Pandemic,” Frontiers in Cellular and Infection Microbiology 12 (2022): 887754, 10.3389/fcimb.2022.887754. 36118039 PMC9471137

[17] E. De Carolis , B. Posteraro , B. Falasca , B. Spruijtenburg , J. F. Meis , and M. Sanguinetti , “The Fourier‐Transform Infrared Spectroscopy‐Based Method as a New Typing Tool for *Candida parapsilosis* Clinical Isolates,” Microbiology Spectrum 11, no. 5 (2023): e0238823, 10.1128/spectrum.02388-23. 37695061 PMC10580913

[18] A. Curtoni , L. Pastrone , M. Cordovana , et al., “Fourier Transform Infrared Spectroscopy Application for *Candida auris* Outbreak Typing in a Referral Intensive Care Unit: Phylogenetic Analysis and Clustering Cut‐Off Definition,” Microorganisms 12, no. 7 (2024): 1312, 10.3390/microorganisms12071312. 39065082 PMC11279149

[19] C. Magrì , E. De Carolis , V. Ivagnes , et al., “‘CLADE‐FINDER’: *Candida auris* Lineage Analysis Determination by Fourier Transform Infrared Spectroscopy and Artificial Neural Networks,” Microorganisms 12, no. 11 (2024): 2153, 10.3390/microorganisms12112153. 39597542 PMC11596196

[20] M. A. Innis , D. H. Gelfand , J. J. Sninsky , and T. J. White , “Amplification and Direct Sequencing of Fungal Ribosomal RNA Genes for Phylogenetics,” in PCR Protocols: A Guide to Methods and Applications (Academic Press, 1990), 315–322.

[21] B. Spruijtenburg , J. N. de Almeida Jr , F. de Camargo Ribeiro , et al., “Multicenter *Candida auris* Outbreak Caused by Azole‐Susceptible Clade IV in Pernambuco, Brazil,” Mycoses 67 (2024): e13752, 10.1111/myc.13752. 38880933

[22] D. E. Carolis , F. Marchionni , M. La Rosa , et al., “Are we Ready for Nosocomial *Candida auris* Infections? Rapid Identification and Antifungal Resistance Detection Using MALDI‐TOF Mass Spectrometry May be the Answer,” Frontiers in Cellular and Infection Microbiology 11 (2021): 645049, 10.3389/fcimb.2021.645049. 33796487 PMC8007968

[23] B. Ali , M. Kumar , P. Kumar , et al., “Sphingolipid Diversity in *Candida auris*: Unraveling Interclade and Drug Resistance Fingerprints,” FEMS Yeast Research 24 (2024): foae008, 10.1093/femsyr/foae008. 38444195 PMC10941814

[24] H. Stieber , L. Junghanns , H. Wilhelm , et al., “The Sphingolipid Inhibitor Myriocin Increases *Candida auris* Susceptibility to Amphotericin B,” Mycoses 67, no. 4 (2024): e13723, 10.1111/myc.13723. 38551121

[25] W. Alfouzan , S. Ahmad , R. Dhar , et al., “Molecular Epidemiology of *Candida auris* Outbreak in a Major Secondary‐Care Hospital in Kuwait,” Journal of Fungi 6, no. 4 (2020): 307, 10.3390/jof6040307. 33233388 PMC7712429

[26] J. Meletiadis , M. Siopi , B. Spruijtenburg , et al., “ *Candida auris* Fungaemia Outbreak in a Tertiary Care Academic Hospital and Emergence of a Pan‐Echinocandin Resistant Isolate, Greece, 2021 to 2023,” Eurosurveillance 29, no. 45 (2024): 2400128, 10.2807/1560-7917.ES.2024.29.45.2400128. 39512169 PMC11544718

