# Supplementary figures and images for: Clade Distinction and Tracking of Clonal Spread by Fourier‐Transform Infrared Spectroscopy in Multicenter Candida (Candidozyma) auris Outbreak
Source: Mycoses. 2025 Jul 4;68(7):e70085. doi: 10.1111/myc.70085 (PMC12232120; doi:10.1111/myc.70085)

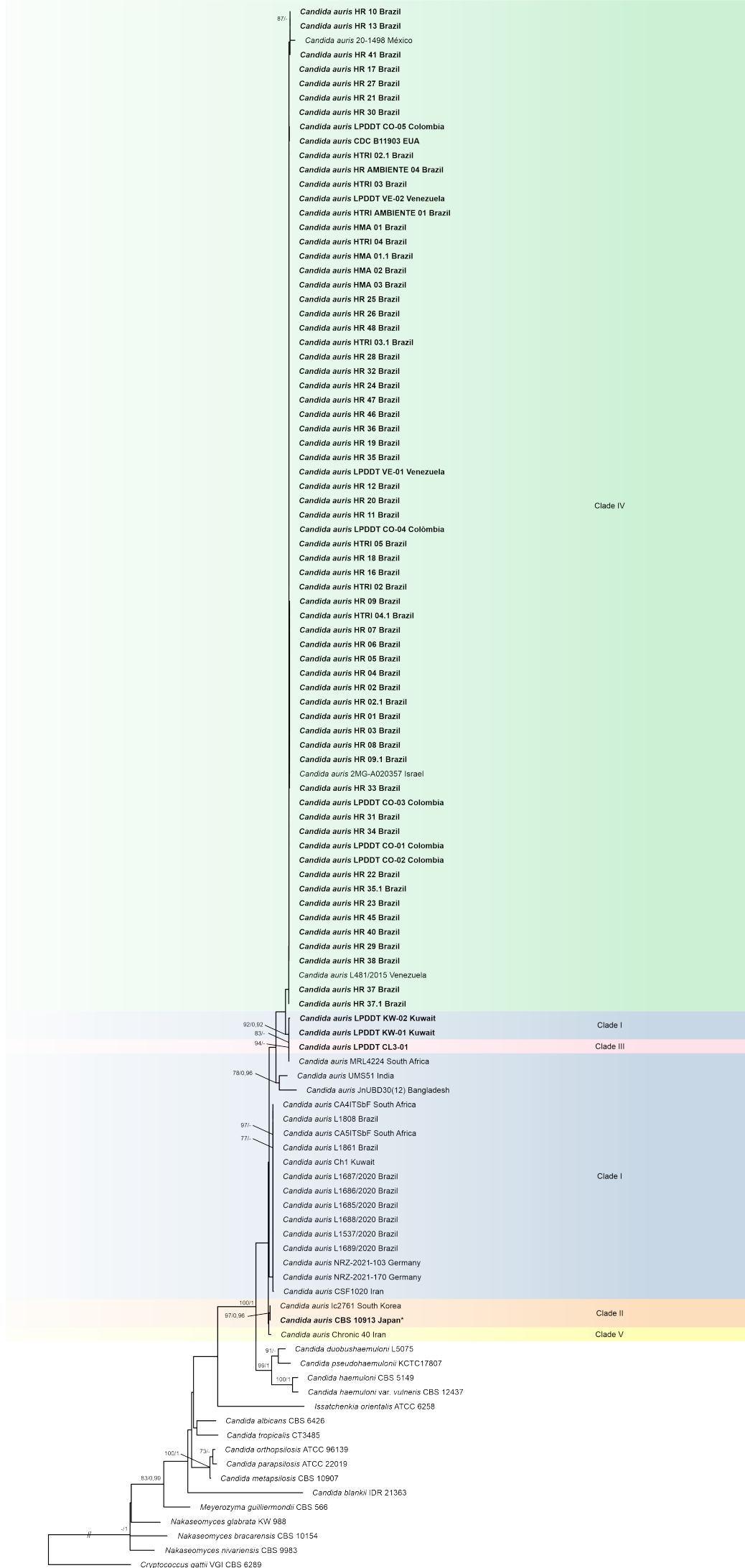

Supplement: Supplementary file 1 — Figure S1. Maximum likelihood (ML) tree obtained using ITS rDNA sequences from Candida species. Study species are in bold. Maximum likelihood (ML‐BS) bootstrap and Bayesian inference posterior probability (BPP) of 70% and 0.90 respectively, are shown near the nodes. The tree was rooted in Cryptococcus gattii VGI (CBS 6289). The bar represents the expected number of replacements per site. The superscript * indicates the ex‐type strain. [file MYC-68-e70085-s004.pdf]
