# Supplementary material for: Clade Distinction and Tracking of Clonal Spread by Fourier‐Transform Infrared Spectroscopy in Multicenter Candida (Candidozyma) auris Outbreak
Source: Mycoses. 2025 Jul 4;68(7):e70085. doi: 10.1111/myc.70085 (PMC12232120; doi:10.1111/myc.70085)
Supplement: Supplementary file 2 — Figure S2. Four snapshots of different angles from the Candida auris clades obtained by 3D scatter plots (LDA 40 PCs, 99.7% variance, target group = isolate ID) showing Clade I in blue, Clade II in red, Clade III in green, and Clade IV in grey. X‐axis displays LD1, y‐axis displays LD2, z‐axis displays LD3. Each dot/shape represents one spectrum. Total of 1582 spectra are displayed. Shapes depict country of origin of the isolated. Graph and legend created with the IR Biotyper software. [file MYC-68-e70085-s001.docx]

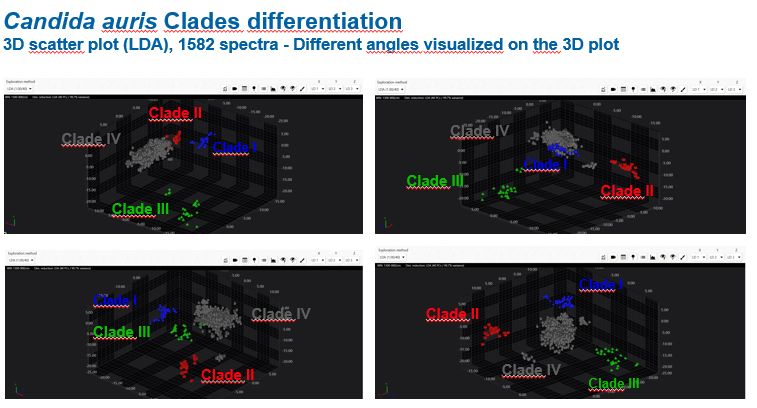


**Figure S2**. Four snapshots of different angles from the *Candida auris* clades obtained by 3D scatter plots (LDA 40 PCs, 99.7% variance, target group = isolate ID) showing Clade I in blue, Clade II in red, Clade III in green, and Clade IV in gray. X-axis displays LD1, y-axis displays LD2, z-axis displays LD3. Each dot/shape represents one spectrum. Total of 1,582 spectra are displayed. Shapes depict country of origin of the isolated. Graph and legend created with the IR Biotyper® software.
