# Supplementary material for: Clade Distinction and Tracking of Clonal Spread by Fourier‐Transform Infrared Spectroscopy in Multicenter Candida (Candidozyma) auris Outbreak
Source: Mycoses. 2025 Jul 4;68(7):e70085. doi: 10.1111/myc.70085 (PMC12232120; doi:10.1111/myc.70085)
Supplement: Supplementary file 3 — Figure S3. 3D scatter plot of Candida auris Clade IV analysed depicting different geographical origins (LDA 40 PCs, 99.6% variance, target group = isolate ID). Blue: Colombia; Grey: Venezuela; Red: Brazil; Green: CDC reference strain. X‐axis displays LD1, y‐axis displays LD2, z‐axis displays LD3, together displaying 55.73% variance. Each dot/shape represents one spectrum. Total of 1470 spectra are displayed. Graph created with the IR Biotyper software. Adjusted splicing method: 1300–800 cm−1 (polysaccharide region), 3000–2800 cm−1 (CH region), 1500–1400 cm−1 (2nd fatty acid). [file MYC-68-e70085-s002.docx]

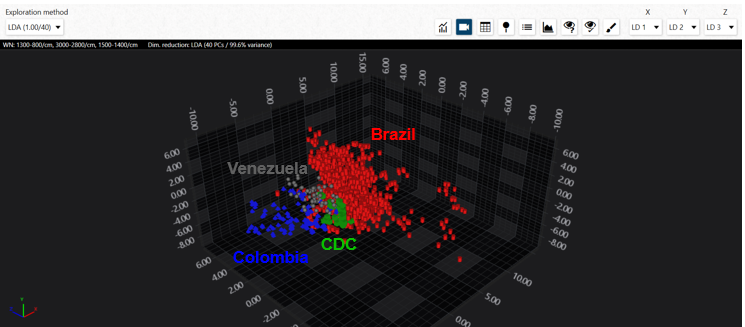


**Figure S3.** 3D scatter plot of *Candida auris* Clade IV analyzed depicting different geographical origins (LDA 40 PCs, 99.6% variance, target group = isolate ID). Blue: Colombia; Gray: Venezuela; Red: Brazil; Green: CDC reference strain. X-axis displays LD1, y-axis displays LD2, z-axis displays LD3, together displaying 55.73% variance. Each dot/shape represents one spectrum. Total of 1,470 spectra are displayed. Graph created with the IR Biotyper® software. Adjusted splicing method: 1300-800 cm^-1^ (polysaccharide region) + 3000 – 2800 cm^-1^ (CH region) + 1500-1400 cm^-1^ (2^nd^ fatty acid).
