# Supplementary material for: Clade Distinction and Tracking of Clonal Spread by Fourier‐Transform Infrared Spectroscopy in Multicenter Candida (Candidozyma) auris Outbreak
Source: Mycoses. 2025 Jul 4;68(7):e70085. doi: 10.1111/myc.70085 (PMC12232120; doi:10.1111/myc.70085)
Supplement: Supplementary file 4 — Figure S4. 3D scatter plot of Candida auris Clade IV from the state of Pernambuco, Brazil, depicting patient isolates originate from 3 different hospitals (LDA 40 PCs, 99.6% variance, target group = isolate ID). Green: HMA; Blue: HR, Yellow: HTRI. X‐axis displays LD1, y‐axis displays LD2, z‐axis displays LD3, together displaying 58.69% variance. Each dot/shape represents one spectrum. Total of 1279 spectra are displayed. Bottom left of the figure is 2D visualisation of Pernambuco subset Candida auris strains. Graph created with the IR Biotyper software. Adjusted splicing method: 1300–800 cm−1 (polysaccharide region) + 3000–2800 cm−1 (CH region) + 1500–1400 cm−1 (2nd fatty acid). [file MYC-68-e70085-s005.docx]

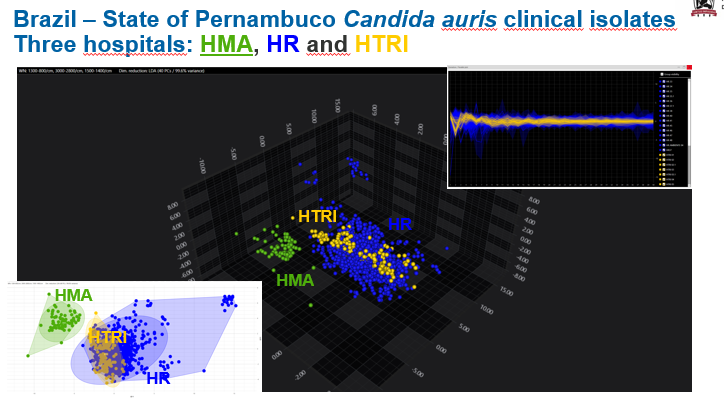


**Figure S4**. 3D scatter plot of *Candida auris* Clade IV from the state of Pernambuco, Brazil, depicting patient isolates originate from 3 different hospitals (LDA 40 PCs, 99.6% variance, target group = isolate ID). Green: HMA; Blue: HR, Yellow: HTRI. X-axis displays LD1, y-axis displays LD2, z-axis displays LD3, together displaying 58.69% variance. Each dot/shape represents one spectrum. Total of 1,279 spectra are displayed. Bottom left of the figure is 2D visualization of Pernambuco subset *Candida auris* strains. Graph created with the IR Biotyper® software. Adjusted splicing method: 1300-800 cm^-1^ (polysaccharide region) + 3000 – 2800 cm^-1^ (CH region) + 1500-1400 cm^-1^ (2^nd^ fatty acid).
