# Supplementary material for: Clade Distinction and Tracking of Clonal Spread by Fourier‐Transform Infrared Spectroscopy in Multicenter Candida (Candidozyma) auris Outbreak
Source: Mycoses. 2025 Jul 4;68(7):e70085. doi: 10.1111/myc.70085 (PMC12232120; doi:10.1111/myc.70085)
Supplement: Supplementary file 5 — Figure S5. STR genotypes of Brazilian C. auris isolates typed with five multiplex PCRs, M2, M3‐I, M3‐II, M3‐III, and M9, which amplify 14 STR targets with repeat sizes of 2, 3, or 9 nucleotides. Cluster analysis displayed that clinical strains from HMA formed a distinct cluster in the end of dendrogram. [file MYC-68-e70085-s003.pptx]

## Slide 1
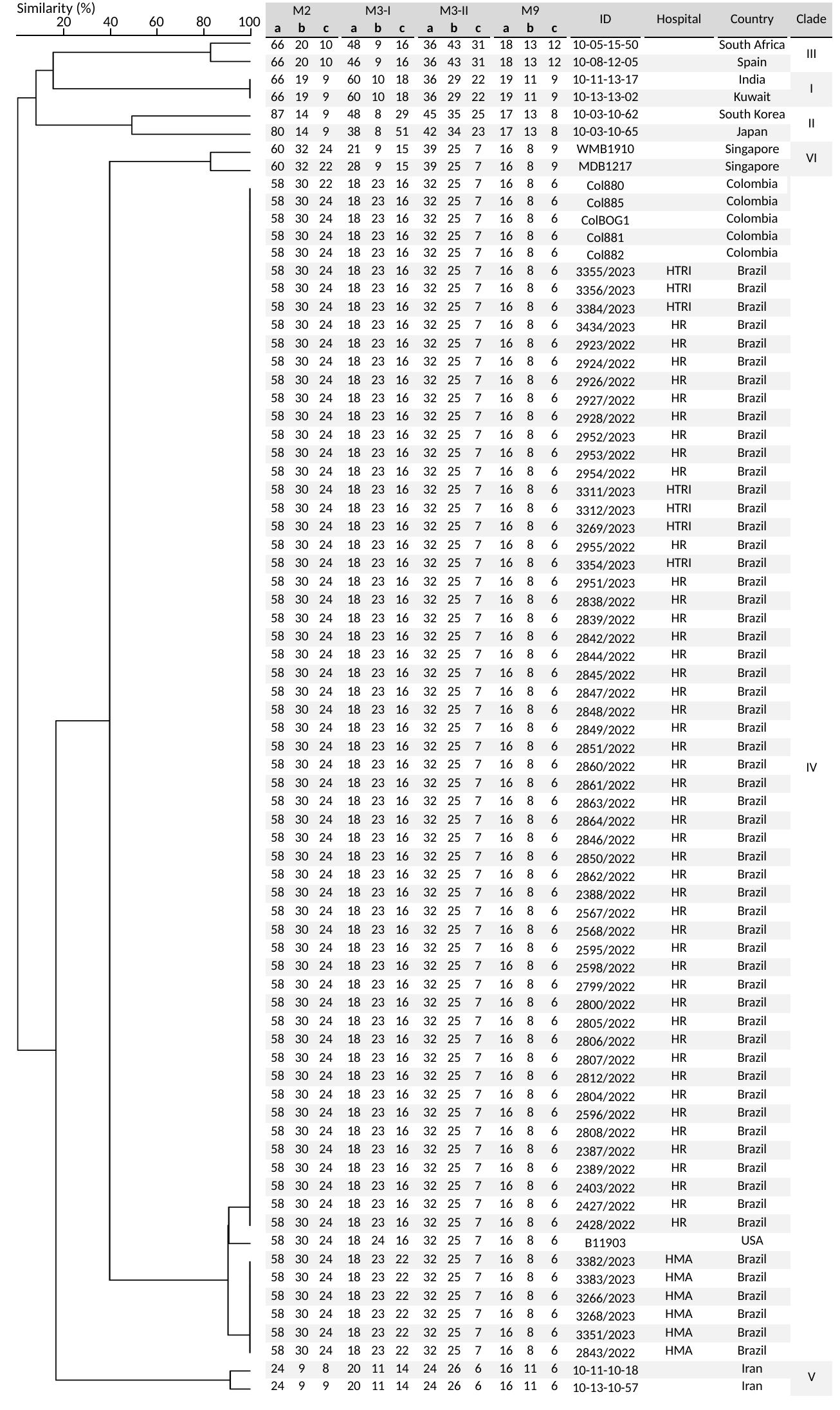

Similarity (%)
| M2 | | | | M3-I | | | | M3-II | | | | M9 | | | | ID | | Hospital | | Country | | Clade |
| --- | --- | --- | --- | --- | --- | --- | --- | --- | --- | --- | --- | --- | --- | --- | --- | --- | --- | --- | --- | --- | --- | --- |
| a | b | c | | a | b | c | | a | b | c | | a | b | c | | | | | | | | |
| 66 | 20 | 10 | | 48 | 9 | 16 | | 36 | 43 | 31 | | 18 | 13 | 12 | | 10-05-15-50 | | | | South Africa | | III |
| 66 | 20 | 10 | | 46 | 9 | 16 | | 36 | 43 | 31 | | 18 | 13 | 12 | | 10-08-12-05 | | | | Spain | | |
| 66 | 19 | 9 | | 60 | 10 | 18 | | 36 | 29 | 22 | | 19 | 11 | 9 | | 10-11-13-17 | | | | India | | I |
| 66 | 19 | 9 | | 60 | 10 | 18 | | 36 | 29 | 22 | | 19 | 11 | 9 | | 10-13-13-02 | | | | Kuwait | | |
| 87 | 14 | 9 | | 48 | 8 | 29 | | 45 | 35 | 25 | | 17 | 13 | 8 | | 10-03-10-62 | | | | South Korea | | II |
| 80 | 14 | 9 | | 38 | 8 | 51 | | 42 | 34 | 23 | | 17 | 13 | 8 | | 10-03-10-65 | | | | Japan | | |
| 60 | 32 | 24 | | 21 | 9 | 15 | | 39 | 25 | 7 | | 16 | 8 | 9 | | WMB1910 | | | | Singapore | | VI |
| 60 | 32 | 22 | | 28 | 9 | 15 | | 39 | 25 | 7 | | 16 | 8 | 9 | | MDB1217 | | | | Singapore | | |
| 58 | 30 | 22 | | 18 | 23 | 16 | | 32 | 25 | 7 | | 16 | 8 | 6 | | Col880 | | | | Colombia | | IV |
| 58 | 30 | 24 | | 18 | 23 | 16 | | 32 | 25 | 7 | | 16 | 8 | 6 | | Col885 | | | | Colombia | | |
| 58 | 30 | 24 | | 18 | 23 | 16 | | 32 | 25 | 7 | | 16 | 8 | 6 | | ColBOG1 | | | | Colombia | | |
| 58 | 30 | 24 | | 18 | 23 | 16 | | 32 | 25 | 7 | | 16 | 8 | 6 | | Col881 | | | | Colombia | | |
| 58 | 30 | 24 | | 18 | 23 | 16 | | 32 | 25 | 7 | | 16 | 8 | 6 | | Col882 | | | | Colombia | | |
| 58 | 30 | 24 | | 18 | 23 | 16 | | 32 | 25 | 7 | | 16 | 8 | 6 | | 3355/2023 | | HTRI | | Brazil | | |
| 58 | 30 | 24 | | 18 | 23 | 16 | | 32 | 25 | 7 | | 16 | 8 | 6 | | 3356/2023 | | HTRI | | Brazil | | |
| 58 | 30 | 24 | | 18 | 23 | 16 | | 32 | 25 | 7 | | 16 | 8 | 6 | | 3384/2023 | | HTRI | | Brazil | | |
| 58 | 30 | 24 | | 18 | 23 | 16 | | 32 | 25 | 7 | | 16 | 8 | 6 | | 3434/2023 | | HR | | Brazil | | |
| 58 | 30 | 24 | | 18 | 23 | 16 | | 32 | 25 | 7 | | 16 | 8 | 6 | | 2923/2022 | | HR | | Brazil | | |
| 58 | 30 | 24 | | 18 | 23 | 16 | | 32 | 25 | 7 | | 16 | 8 | 6 | | 2924/2022 | | HR | | Brazil | | |
| 58 | 30 | 24 | | 18 | 23 | 16 | | 32 | 25 | 7 | | 16 | 8 | 6 | | 2926/2022 | | HR | | Brazil | | |
| 58 | 30 | 24 | | 18 | 23 | 16 | | 32 | 25 | 7 | | 16 | 8 | 6 | | 2927/2022 | | HR | | Brazil | | |
| 58 | 30 | 24 | | 18 | 23 | 16 | | 32 | 25 | 7 | | 16 | 8 | 6 | | 2928/2022 | | HR | | Brazil | | |
| 58 | 30 | 24 | | 18 | 23 | 16 | | 32 | 25 | 7 | | 16 | 8 | 6 | | 2952/2023 | | HR | | Brazil | | |
| 58 | 30 | 24 | | 18 | 23 | 16 | | 32 | 25 | 7 | | 16 | 8 | 6 | | 2953/2022 | | HR | | Brazil | | |
| 58 | 30 | 24 | | 18 | 23 | 16 | | 32 | 25 | 7 | | 16 | 8 | 6 | | 2954/2022 | | HR | | Brazil | | |
| 58 | 30 | 24 | | 18 | 23 | 16 | | 32 | 25 | 7 | | 16 | 8 | 6 | | 3311/2023 | | HTRI | | Brazil | | |
| 58 | 30 | 24 | | 18 | 23 | 16 | | 32 | 25 | 7 | | 16 | 8 | 6 | | 3312/2023 | | HTRI | | Brazil | | |
| 58 | 30 | 24 | | 18 | 23 | 16 | | 32 | 25 | 7 | | 16 | 8 | 6 | | 3269/2023 | | HTRI | | Brazil | | |
| 58 | 30 | 24 | | 18 | 23 | 16 | | 32 | 25 | 7 | | 16 | 8 | 6 | | 2955/2022 | | HR | | Brazil | | |
| 58 | 30 | 24 | | 18 | 23 | 16 | | 32 | 25 | 7 | | 16 | 8 | 6 | | 3354/2023 | | HTRI | | Brazil | | |
| 58 | 30 | 24 | | 18 | 23 | 16 | | 32 | 25 | 7 | | 16 | 8 | 6 | | 2951/2023 | | HR | | Brazil | | |
| 58 | 30 | 24 | | 18 | 23 | 16 | | 32 | 25 | 7 | | 16 | 8 | 6 | | 2838/2022 | | HR | | Brazil | | |
| 58 | 30 | 24 | | 18 | 23 | 16 | | 32 | 25 | 7 | | 16 | 8 | 6 | | 2839/2022 | | HR | | Brazil | | |
| 58 | 30 | 24 | | 18 | 23 | 16 | | 32 | 25 | 7 | | 16 | 8 | 6 | | 2842/2022 | | HR | | Brazil | | |
| 58 | 30 | 24 | | 18 | 23 | 16 | | 32 | 25 | 7 | | 16 | 8 | 6 | | 2844/2022 | | HR | | Brazil | | |
| 58 | 30 | 24 | | 18 | 23 | 16 | | 32 | 25 | 7 | | 16 | 8 | 6 | | 2845/2022 | | HR | | Brazil | | |
| 58 | 30 | 24 | | 18 | 23 | 16 | | 32 | 25 | 7 | | 16 | 8 | 6 | | 2847/2022 | | HR | | Brazil | | |
| 58 | 30 | 24 | | 18 | 23 | 16 | | 32 | 25 | 7 | | 16 | 8 | 6 | | 2848/2022 | | HR | | Brazil | | |
| 58 | 30 | 24 | | 18 | 23 | 16 | | 32 | 25 | 7 | | 16 | 8 | 6 | | 2849/2022 | | HR | | Brazil | | |
| 58 | 30 | 24 | | 18 | 23 | 16 | | 32 | 25 | 7 | | 16 | 8 | 6 | | 2851/2022 | | HR | | Brazil | | |
| 58 | 30 | 24 | | 18 | 23 | 16 | | 32 | 25 | 7 | | 16 | 8 | 6 | | 2860/2022 | | HR | | Brazil | | |
| 58 | 30 | 24 | | 18 | 23 | 16 | | 32 | 25 | 7 | | 16 | 8 | 6 | | 2861/2022 | | HR | | Brazil | | |
| 58 | 30 | 24 | | 18 | 23 | 16 | | 32 | 25 | 7 | | 16 | 8 | 6 | | 2863/2022 | | HR | | Brazil | | |
| 58 | 30 | 24 | | 18 | 23 | 16 | | 32 | 25 | 7 | | 16 | 8 | 6 | | 2864/2022 | | HR | | Brazil | | |
| 58 | 30 | 24 | | 18 | 23 | 16 | | 32 | 25 | 7 | | 16 | 8 | 6 | | 2846/2022 | | HR | | Brazil | | |
| 58 | 30 | 24 | | 18 | 23 | 16 | | 32 | 25 | 7 | | 16 | 8 | 6 | | 2850/2022 | | HR | | Brazil | | |
| 58 | 30 | 24 | | 18 | 23 | 16 | | 32 | 25 | 7 | | 16 | 8 | 6 | | 2862/2022 | | HR | | Brazil | | |
| 58 | 30 | 24 | | 18 | 23 | 16 | | 32 | 25 | 7 | | 16 | 8 | 6 | | 2388/2022 | | HR | | Brazil | | |
| 58 | 30 | 24 | | 18 | 23 | 16 | | 32 | 25 | 7 | | 16 | 8 | 6 | | 2567/2022 | | HR | | Brazil | | |
| 58 | 30 | 24 | | 18 | 23 | 16 | | 32 | 25 | 7 | | 16 | 8 | 6 | | 2568/2022 | | HR | | Brazil | | |
| 58 | 30 | 24 | | 18 | 23 | 16 | | 32 | 25 | 7 | | 16 | 8 | 6 | | 2595/2022 | | HR | | Brazil | | |
| 58 | 30 | 24 | | 18 | 23 | 16 | | 32 | 25 | 7 | | 16 | 8 | 6 | | 2598/2022 | | HR | | Brazil | | |
| 58 | 30 | 24 | | 18 | 23 | 16 | | 32 | 25 | 7 | | 16 | 8 | 6 | | 2799/2022 | | HR | | Brazil | | |
| 58 | 30 | 24 | | 18 | 23 | 16 | | 32 | 25 | 7 | | 16 | 8 | 6 | | 2800/2022 | | HR | | Brazil | | |
| 58 | 30 | 24 | | 18 | 23 | 16 | | 32 | 25 | 7 | | 16 | 8 | 6 | | 2805/2022 | | HR | | Brazil | | |
| 58 | 30 | 24 | | 18 | 23 | 16 | | 32 | 25 | 7 | | 16 | 8 | 6 | | 2806/2022 | | HR | | Brazil | | |
| 58 | 30 | 24 | | 18 | 23 | 16 | | 32 | 25 | 7 | | 16 | 8 | 6 | | 2807/2022 | | HR | | Brazil | | |
| 58 | 30 | 24 | | 18 | 23 | 16 | | 32 | 25 | 7 | | 16 | 8 | 6 | | 2812/2022 | | HR | | Brazil | | |
| 58 | 30 | 24 | | 18 | 23 | 16 | | 32 | 25 | 7 | | 16 | 8 | 6 | | 2804/2022 | | HR | | Brazil | | |
| 58 | 30 | 24 | | 18 | 23 | 16 | | 32 | 25 | 7 | | 16 | 8 | 6 | | 2596/2022 | | HR | | Brazil | | |
| 58 | 30 | 24 | | 18 | 23 | 16 | | 32 | 25 | 7 | | 16 | 8 | 6 | | 2808/2022 | | HR | | Brazil | | |
| 58 | 30 | 24 | | 18 | 23 | 16 | | 32 | 25 | 7 | | 16 | 8 | 6 | | 2387/2022 | | HR | | Brazil | | |
| 58 | 30 | 24 | | 18 | 23 | 16 | | 32 | 25 | 7 | | 16 | 8 | 6 | | 2389/2022 | | HR | | Brazil | | |
| 58 | 30 | 24 | | 18 | 23 | 16 | | 32 | 25 | 7 | | 16 | 8 | 6 | | 2403/2022 | | HR | | Brazil | | |
| 58 | 30 | 24 | | 18 | 23 | 16 | | 32 | 25 | 7 | | 16 | 8 | 6 | | 2427/2022 | | HR | | Brazil | | |
| 58 | 30 | 24 | | 18 | 23 | 16 | | 32 | 25 | 7 | | 16 | 8 | 6 | | 2428/2022 | | HR | | Brazil | | |
| 58 | 30 | 24 | | 18 | 24 | 16 | | 32 | 25 | 7 | | 16 | 8 | 6 | | B11903 | | | | USA | | |
| 58 | 30 | 24 | | 18 | 23 | 22 | | 32 | 25 | 7 | | 16 | 8 | 6 | | 3382/2023 | | HMA | | Brazil | | |
| 58 | 30 | 24 | | 18 | 23 | 22 | | 32 | 25 | 7 | | 16 | 8 | 6 | | 3383/2023 | | HMA | | Brazil | | |
| 58 | 30 | 24 | | 18 | 23 | 22 | | 32 | 25 | 7 | | 16 | 8 | 6 | | 3266/2023 | | HMA | | Brazil | | |
| 58 | 30 | 24 | | 18 | 23 | 22 | | 32 | 25 | 7 | | 16 | 8 | 6 | | 3268/2023 | | HMA | | Brazil | | |
| 58 | 30 | 24 | | 18 | 23 | 22 | | 32 | 25 | 7 | | 16 | 8 | 6 | | 3351/2023 | | HMA | | Brazil | | |
| 58 | 30 | 24 | | 18 | 23 | 22 | | 32 | 25 | 7 | | 16 | 8 | 6 | | 2843/2022 | | HMA | | Brazil | | |
| 24 | 9 | 8 | | 20 | 11 | 14 | | 24 | 26 | 6 | | 16 | 11 | 6 | | 10-11-10-18 | | | | Iran | | V |
| 24 | 9 | 9 | | 20 | 11 | 14 | | 24 | 26 | 6 | | 16 | 11 | 6 | | 10-13-10-57 | | | | Iran | | |
20
60
80
40
100
