# Supplementary material for: Clade Distinction and Tracking of Clonal Spread by Fourier‐Transform Infrared Spectroscopy in Multicenter Candida (Candidozyma) auris Outbreak
Source: Mycoses. 2025 Jul 4;68(7):e70085. doi: 10.1111/myc.70085 (PMC12232120; doi:10.1111/myc.70085)
Supplement: Supplementary file 6 — Table S1. Overview of Candida spp. used in this study. All strains were used to construct the ITS phylogenetic analysis. The strains highlighted in bold were used in Fourier‐Transform Infrared Spectroscopy analysis. [file MYC-68-e70085-s006.docx]

| Table S1: Overview of *Candida* spp. used in this study. All strains were used to construct the ITS phylogenetic analysis. The strains highlighted in bold were used in Fourier-Transform Infrared Spectroscopy analysis. | | | | | |  |
| --- | --- | --- | --- | --- | --- | --- |
| Specie | **Strain** | **Isolation source** | **Clade** | **Country** | **GenBank number ITS** | |
| *Candida auris* | L1537 | Catheter tip | I | Brazil | MW463338.1 | |
| *Candida auris* | L1861 | Bloodstream | I | Brazil | MW989535.1 | |
| *Candida auris* | L1686 | Catheter tip | I | Brazil | MW463340.1 | |
| *Candida auris* | L1687 | Bed rails | I | Brazil | MW463341.1 | |
| *Candida auris* | L1688 | Mechanical ventilator/infusion pump/vital signs monitor | I | Brazil | MW463342.1 | |
| *Candida auris* | L1689 | Thermometer | I | Brazil | MW463343.1 | |
| *Candida auris* | L1808 | Axillae swab | I | Brazil | MW989534.1 | |
| *Candida auris* | L1685 | Bloodstream | I | Brazil | MW463339.1 | |
| *Candida auris* | CSF1020 | cerebrospinal fluid | I | Iran | MZ853742.1 | |
| *Candida auris* | NRZ_2021_103 | Urine | I | Germany | OK310884.1 | |
| *Candida auris* | NRZ_2021_170 | Tracheal lavage | I | Germany | OK310890.1 | |
| *Candida auris* | UMS51 | Human | I | India | MN542752.1 | |
| *Candida auris* | CH1 | Bloodstream | I | Kuwait | HE797772.1 | |
| *Candida auris* | Jnubd30(12) | Human | I | Bangladesh | MH427523.1 | |
| *Candida auris* | MRL4224 | Human | I | South Africa | KY657045.1 | |
| *Candida auris* | CA4ITSbF | Unknown | I | South Arabia | MW039131.1 | |
| *Candida auris* | CA5ITSbF | Unknown | I | South Arabia | MW039132.1 | |
| *Candida auris* | **LPDDT KW-01** | **Human** | **I** | **Kuwait** | **PV059361** | |
| *Candida auris* | **LPDDT KW-02** | **Human** | **I** | **Kuwait** | **PV059362** | |
| *Candida auris* | **CBS 10913 = B11220** | **Ear canal** | **II** | **Japan** | **NR154998.1** | |
| *Candida auris* | IC2761 | Human | II | South Korea | MK308751.1 | |
| *Candida auris* | **LPDDT CL3-01** | **Unknown** | **III** | **Spain** | **PV059363** | |
| *Candida auris* | L48/2015 | Bloodstream | IV | Venezuela | KT305984 | |
| *Candida auris* | 20-1498 | Bloodstream | IV | México | MT704968.1 | |
| *Candida auris* | 2MG-A0203-57 | Human | IV | Israel | MN243082.1 | |
| *Candida auris* | **HR 01 = CRA 09 = URM 8610** | **Urine** | **IV** | **Brazil** | **ON256389.1** | |
| *Candida auris* | **HR 02 = CRA 07 = URM 8611** | **Urine** | **IV** | **Brazil** | **ON256397.1** | |
| *Candida auris* | **HR 02.1 = CRA 10 = URM 8612** | **Urine** | **IV** | **Brazil** | **ON256398.1** | |
| *Candida auris* | **HR 03 = CRA 11 = URM 8613** | **Axillary/inguinal swab** | **IV** | **Brazil** | **ON256390.1** | |
| *Candida auris* | **HR 04 = CRA 08 = URM 8614** | **Axillary/inguinal swab** | **IV** | **Brazil** | **ON256391.1** | |
| *Candida auris* | **HR 05 = CRA06 = URM 8615** | **Axillary/inguinal swab** | **IV** | **Brazil** | **ON256396.1** | |
| *Candida auris* | **HR 06 = CRA02 = URM 8616** | **Axillary/inguinal swab** | **IV** | **Brazil** | **ON256393.1** | |
| *Candida auris* | **HR 07 = CRA05 = URM 8617** | **Axillary/inguinal swab** | **IV** | **Brazil** | **ON256395.1** | |
| *Candida auris* | **HR 08 = CRA04 = URM 8618** | **Axillary/inguinal swab** | **IV** | **Brazil** | **ON256388.1** | |
| *Candida auris* | **HR 09 = CRA03 = URM 8619** | **Axillary/inguinal swab** | **IV** | **Brazil** | **ON256394.1** | |
| *Candida auris* | **HR 09.1 = CRA01 = URM 8635** | **Axillary/inguinal swab** | **IV** | **Brazil** | **ON256392.1** | |
| *Candida auris* | **HR 10** | **Axillary/inguinal swab** | **IV** | **Brazil** | **PV059305** | |
| *Candida auris* | **HR 11** | **Axillary/inguinal swab** | **IV** | **Brazil** | **PV059306** | |
| *Candida auris* | **HR 12** | **Axillary/inguinal swab** | **IV** | **Brazil** | **PV059307** | |
| *Candida auris* | **HR 13** | **Axillary/inguinal swab** | **IV** | **Brazil** | **PV059308** | |
| *Candida auris* | **HR 16** | **Axillary/inguinal swab** | **IV** | **Brazil** | **PV059309** | |
| *Candida auris* | **HR 17** | **Axillary/inguinal swab** | **IV** | **Brazil** | **PV059310** | |
| *Candida auris* | **HR 18** | **Axillary/inguinal swab** | **IV** | **Brazil** | **PV059311** | |
| *Candida auris* | **HR 19** | **Axillary/inguinal swab** | **IV** | **Brazil** | **PV059312** | |
| *Candida auris* | **HR 20** | **Axillary/inguinal swab** | **IV** | **Brazil** | **PV059313** | |
| *Candida auris* | **HR 21** | **Axillary/inguinal swab** | **IV** | **Brazil** | **PV059314** | |
| *Candida auris* | **HR 22** | **Axillary/inguinal swab** | **IV** | **Brazil** | **PV059315** | |
| *Candida auris* | **HR 23** | **Axillary/inguinal swab** | **IV** | **Brazil** | **PV059316** | |
| *Candida auris* | **HR 24** | **Axillary/inguinal swab** | **IV** | **Brazil** | **PV059317** | |
| *Candida auris* | **HR 25** | **Axillary/inguinal swab** | **IV** | **Brazil** | **PV059318** | |
| *Candida auris* | **HR 26** | **Axillary/inguinal swab** | **IV** | **Brazil** | **PV059319** | |
| *Candida auris* | **HR 27** | **Axillary/inguinal swab** | **IV** | **Brazil** | **PV059320** | |
| *Candida auris* | **HR 28** | **Axillary/inguinal swab** | **IV** | **Brazil** | **PV059321** | |
| *Candida auris* | **HR 29** | **Axillary/inguinal swab** | **IV** | **Brazil** | **PV059322** | |
| *Candida auris* | **HR 30** | **Axillary/inguinal swab** | **IV** | **Brazil** | **PV059323** | |
| *Candida auris* | **HR 31** | **Axillary/inguinal swab** | **IV** | **Brazil** | **PV059324** | |
| *Candida auris* | **HR 32** | **Axillary/inguinal swab** | **IV** | **Brazil** | **PV059325** | |
| *Candida auris* | **HR 33** | **Axillary/inguinal swab** | **IV** | **Brazil** | **PV059326** | |
| *Candida auris* | **HR 34** | **Axillary/inguinal swab** | **IV** | **Brazil** | **PV059327** | |
| *Candida auris* | **HR 35** | **Axillary/inguinal swab** | **IV** | **Brazil** | **PV059328** | |
| *Candida auris* | **HR 35.1** | **Urine** | **IV** | **Brazil** | **PV059329** | |
| *Candida auris* | **HR 36** | **Axillary/inguinal swab** | **IV** | **Brazil** | **PV059330** | |
| *Candida auris* | **HR 37** | **Bloodstream** | **IV** | **Brazil** | **PV059331** | |
| *Candida auris* | **HR 37.1** | **Catheter tip** | **IV** | **Brazil** | **PV059332** | |
| *Candida auris* | **HR 38** | **Axillary/inguinal swab** | **IV** | **Brazil** | **PV059333** | |
| *Candida auris* | **HR 40** | **Axillary/inguinal swab** | **IV** | **Brazil** | **PV059334** | |
| *Candida auris* | **HR 41** | **Axillary/inguinal swab** | **IV** | **Brazil** | **PV059335** | |
| *Candida auris* | **HR 45** | **Axillary/inguinal swab** | **IV** | **Brazil** | **PV059336** | |
| *Candida auris* | **HR 46** | **Axillary/inguinal swab** | **IV** | **Brazil** | **PV059337** | |
| *Candida auris* | **HR 47** | **Axillary/inguinal swab** | **IV** | **Brazil** | **PV059338** | |
| *Candida auris* | **HR 48** | **Axillary/inguinal swab** | **IV** | **Brazil** | **PV059339** | |
| *Candida auris* | **HR AMB 04** | **Bed** | **IV** | **Brazil** | **PV059340** | |
| *Candida auris* | **HTRI 02** | **Urine** | **IV** | **Brazil** | **PV059341** | |
| *Candida auris* | **HTRI 02.1** | **Urine** | **IV** | **Brazil** | **PV059342** | |
| *Candida auris* | **HTRI 03** | **Axillary/inguinal swab** | **IV** | **Brazil** | **PV059343** | |
| *Candida auris* | **HTRI 03.1** | **Axillary/inguinal swab** | **IV** | **Brazil** | **PV059344** | |
| *Candida auris* | **HTRI 04** | **Axillary/inguinal swab** | **IV** | **Brazil** | **PV059345** | |
| *Candida auris* | **HTRI 04.1** | **Axillary/inguinal swab** | **IV** | **Brazil** | **PV059346** | |
| *Candida auris* | **HTRI 05** | **Axillary/inguinal swab** | **IV** | **Brazil** | **PV059347** | |
| *Candida auris* | TRI AMB 01 | Unknown | IV | Brazil | PV059348 | |
| *Candida auris* | **HMA 01** | **Human** | **IV** | **Brazil** | **PV059349** | |
| *Candida auris* | **HMA 01.1** | **Axillary/inguinal swab** | **IV** | **Brazil** | **PV059350** | |
| *Candida auris* | **HMA 02** | **Axillary/inguinal swab** | **IV** | **Brazil** | **PV059351** | |
| *Candida auris* | **HMA 03** | **Catheter tip** | **IV** | **Brazil** | **PV059352** | |
| *Candida auris* | **LPDDT VE-01** | **Human** | **IV** | **Venezuela** | **PV059353** | |
| *Candida auris* | **LPDDT VE-02** | **Human** | **IV** | **Venezuela** | **PV059354** | |
| *Candida auris* | **LPDDT CO-01** | **Human** | **IV** | **Colombia** | **PV059355** | |
| *Candida auris* | **LPDDT CO-02** | **Human** | **IV** | **Colombia** | **PV059356** | |
| *Candida auris* | **LPDDT CO-03** | **Human** | **IV** | **Colombia** | **PV059357** | |
| *Candida auris* | LPDDT CO-04 | Human | IV | Colombia | PV059358 | |
| *Candida auris* | LPDDT CO-05 | Human | IV | Colombia | PV059359 | |
| *Candida auris* | **CDC B11903** | **Unknown** | **IV** | **USA** | **PV059360** | |
| *Candida auris* | Chronic 40 | Ear canal | V | Iran | MZ389242.1 | |
| *Candida albicans* | CBS 6426 | - | - | - | KY101918.1 | |
| *Candida tropicalis* | CT 348-5 | - | - | - | MW358908.1 | |
| *Candida parapsilosis* | ATCC 22019 | - | - | - | NR130673.1 | |
| *Candida metapsilosis* | CBS 10907 | - | - | - | NR165186.1 | |
| *Candida orthopsilosis* | ATCC 96139 | - | - | - | NR130661.1 | |
| *Candida haemuloni* | CBS 5149 | - | - | - | AY500375.1 | |
| *Candida haemuloni* var. *vulneris* | CBS 12437 | - | - | - | KY102119.1 | |
| *Candida duobushaemuloni* | L5075 | - | - | - | KC408994.1 | |
| *Candida pseudohaemulonii* | KCTC17807 | - | - | - | JX459678.1 | |
| *Candida blankii* | IDR21_363 | - | - | - | OL697232.1 | |
| *Nakaseomyces glabrata* | Kw998 | - | - | - | AM492797.1 | |
| *Nakaseomyces bracariensis* | CBS 10154 | - | - | - | MW284423.1 | |
| *Nakaseomyces nivariensis* | CBS 9983 | - | - | - | NR_077073.1 | |
| *Meyerozyma guilliermondii* | CBS 566 | - | - | - | EU568911.1 | |
| *Issatchenkia orientalis* | ATCC 6258 | - | - | - | NR_131315.1 | |
| *Cryptococcus gattii* VGI | CBS 6289 | - | - | - | NR_165941.1 | |
